# Time-Dependent Density Functional Theory Calculations of N- and S-Doped TiO_2_ Nanotube for Water-Splitting Applications

**DOI:** 10.3390/nano11112900

**Published:** 2021-10-29

**Authors:** Yin-Pai Lin, Inta Isakoviča, Aleksejs Gopejenko, Anna Ivanova, Aleksandrs Začinskis, Roberts I. Eglitis, Pavel N. D’yachkov, Sergei Piskunov

**Affiliations:** 1 Institute of Solid State Physics, University of Latvia, 8 Kengaraga Str., LV-1063 Riga, Latvia; in-bai.lin@cfi.lu.lv (Y.-P.L.); intai@cfi.lu.lv (I.I.); agopejen@cfi.lu.lv (A.G.); ann.che@cfi.lu.lv (A.I.); aleksandrs.zacinskis@cfi.lu.lv (A.Z.); rieglitis@cfi.lu.lv (R.I.E.); 2 Kurnakov Institute of General and Inorganic Chemistry of the Russian Academy of Sciences, Leninskii Pr. 31, 119991 Moscow, Russia; p_dyachkov@rambler.ru

**Keywords:** TiO_2_ nanotube, photocatalyst, time-dependent density functional theory, absorption spectra, transition contribution maps

## Abstract

On the basis of time-dependent density functional theory (TD-DFT) we performed first-principle calculations to predict optical properties and transition states of pristine, N- and S-doped, and N+S-codoped anatase TiO${}_{2}$ nanotubes of 1 nm-diameter. The host O atoms of the pristine TiO${}_{2}$ nanotube were substituted by N and S atoms to evaluate the influence of dopants on the photocatalytic properties of hollow titania nanostructures. The charge transition mechanism promoted by dopants positioned in the nanotube wall clearly demonstrates the constructive and destructive contributions to photoabsorption by means of calculated transition contribution maps. Based on the results of our calculations, we predict an increased visible-light-driven photoresponse in N- and S-doped and the N+S-codoped TiO${}_{2}$ nanotubes, enhancing the efficiency of hydrogen production in water-splitting applications.

## 1. Introduction

Use of photodriven semiconductor-based catalyst is a promising route for energy production from sunlight. One of the main directions in this respect is utilization of the solar power for producing hydrogen fuel through the photocatalytic water-splitting reaction. Among all photocatalytic materials, titania (TiO${}_{2}$) has been widely investigated since it was described for the first time by Fujishima and Honda [1]. The band gap width of TiO${}_{2}$ (from 3.0 to 3.2 eV) allows efficient water-splitting under UV irradiation due to the positions of band edges—valence band maximum (VBM) of TiO${}_{2}$ is located below the oxidation potential of water, while its conduction band minimum (CBM) is located above the proton reduction potential in many synthesized TiO${}_{2}$-based compounds [2,3,4,5,6,7,8,9,10,11,12]. However, the relatively wide band gap of undoped TiO${}_{2}$ leads to relatively poor efficiency of light absorption in the visible range of solar spectrum.

The total efficiency of the photocatalytic process strongly depends on charge transport and charge separation processes taking place in photocatalytic materials. In this respect, one-dimensional hollow nanostructures exhibit unique and advantageous features in comparison with three-dimensional and two-dimensional phases of photocatalytic materials [13]. However, the quantum confinement effect leads to a slightly increased band gap of nanotubes (NTs). To overcome these drawback, nonmetal dopants (such as N and S) have been proposed for TiO${}_{2}$ NT to induce in-gap states allowing a visible-light-driven photoresponse suitable for water splitting [14,15].

Nowadays, with the tremendous revolution of computation techniques, theoretical calculations based on quantum–chemical methods have become a significant tool to investigate photon-absorption mechanisms in solids. Density functional theory (DFT) and time-dependent density functional theory (TD-DFT) accompanied with the linear combination of atomic orbitals (LCAO) method allow the simulations of up to hundreds-atom structures and provide the reasonable equilibrium between accuracy, computational cost, and agreement with the experiment [16,17,18,19,20,21,22,23,24,25].

In this work, we focus on the theoretical predictions of electronic structure and optical properties of N- and S-doped TiO${}_{2}$ NT. Calculations using both DFT and TD-DFT were performed to analyze the ground state, optical properties, absorption spectra, and to build the transition contribution maps (TCM) of doped TiO${}_{2}$ NT. To predict the atomic composition of doped NT with the most-enhanced absorption properties, the following methodology is adopted: (i) calculations of electronic structure of N- and S-doped TiO${}_{2}$ NT as a function of the dopant concentration; (ii) calculation of the absorption spectra and TCM to analyze the electron and hole transition mechanism in the doped NT; (iii) modeling of the N+S-codoped TiO${}_{2}$ NT to combine the advantage of both N and S dopants to fulfill the criteria necessary for visible-light-driven photocatalytic water splitting.

## 2. Computational Details

Based on the equilibrium geometries of TiO${}_{2}$ NT obtained using CRYSTAL17 package [26], the DFT and TD-DFT calculations were performed using the GPAW computer code [27,28] accompanied with ASE library [29]. Orbital configurations of the valence electrons were used as follows: O(2s${}^{2}$2p${}^{4}$), Ti(3s${}^{2}$3p${}^{6}$3d${}^{2}$4s${}^{2}$), N(2s${}^{2}$2p${}^{3}$), and S(3s${}^{2}$3p${}^{4}$). The default PAW dataset package 0.9.20000 and the double-ζ polarized (dzp) basis sets provided in GPAW package were utilized for all atoms consisting doped NT. Periodic boundary conditions were emposed along the *x* direction of the NT, while the vacuum gap of 15 Å width has been set along its *y*-*z* directions. The complete geometry optimization was performed using the Broyden–Fletcher–Goldfarb–Shanno (BFGS) algorithm. Calculations based on Perdew–Burke–Ernzerhof (PBE) exchange–correlation functional [30] within DFT were carried out to reach the equilibrium positions of all atoms comprising the NT. Optical properties of the relaxed TiO${}_{2}$ NT with and without substitutional atoms (N, S) were calculated using the LCAO time propagation TD-DFT. To perform electronic band structure calculation, the Gritsenko–van Leeuwen–van Lenthe–Baerends functional with the solid-state modification (GLLB-SC) [31] was adopted due to its ability to describe reliably the band gap of TiO${}_{2}$ [32].

In the ground state calculations at LCAO level of theory [33], the electronic structure of NTs was calculated using GLLB-SC functional and coarser grid of 0.25 A. In order to perform the TD-DFT/LCAO time-propagation calculations necessary to obtain the optical absorption spectrum and TCM [16,34], our NT structures were evoked by weak δ-kick (dipole approximation) along *z* direction to simulate the incident light perpendicular to the nanotube wall. A semi-implicit Crank–Nicolson method was used to propagate the TD-DFT/LCAO [16,34]. The time-dependent dipole moments were imposed along the *z* axis to obtain the photoabsorption spectrum by means of Fourier transform [34]. The Gaussian (Lorentzian) spectral broadening with σ (η) corresponding to a full width at half-maximum (FWHM) has been used for time propagation [16,18,34]. In order to reduce the computational cost, the smallest length of the time-step and the total propagation time were adjusted according to the receipts given in Ref. [16]. The total propagation time used in this study was set to 500 time-steps of 20 attoseconds, each within the total propagation time of 10 femtoseconds. The photoabsorption spectra were calculated using the Lorentzian broadening function with η = 0.07 eV.

The complete photoabsorption spectrum S(ω) is composed from spectral constituents built from the Kohn–Sham decomposition of occupied states (electrons) to the unoccupied states (holes) S${}_{occ.}^{unocc.}$(ω). In TCM, the ratio of S${}_{occ.}^{unocc.}$(ω) and S(ω) is illustrated by scattering on Gaussian-broadened two-dimensional surface spanned by the density of states (DOS) drawn above (below) the Fermi level at the vertical and horizontal axes, respectively. The TCM is an illustrative data visualization to observe the superposition of multiple electron and hole transitions for the photo-absorption at corresponding photon energy (wavelength, ω). The transition contribution are indicated by red (positive) and blue (negative) isolines. The positive (negative) transition contribution reveals the constructive (destructive) contribution of S${}_{occ.}^{unocc.}$(ω) to the S(ω) (photoabsorption). For more details of LCAO time propagation in TD-DFT and TCM, please see Refs. [16,17,18,34,35]. VESTA software package [36] was used to visualize the atomic structure and positions of pseudowavefunction of NTs under study. Python packages NumPy [37] and Matplotlib [38] were utilized to analyze computed data and to produce the figures.

## 3. Results and Discussion

### 3.1. Pristine TiO${}_{2}$ NT

**Figure 1:**
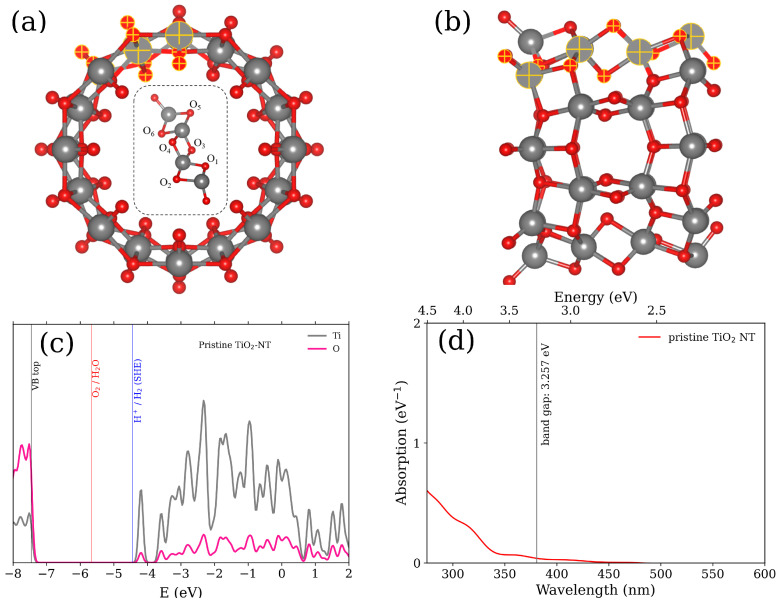
Schematic illustration of the pristine (8,0) anatase (101) TiO${}_{2}$ NT with the diameter of 0.933 nm: (**a**) front view of the NT, and (**b**) side view of the NT. Ti atoms are shown as gray balls, oxygens are shown as red balls. The inset in (**a**) shows the unit cell of the TiO${}_{2}$ NT repeated 8 times by rototranslational symmetry. The numbered O atoms denote the doping sites. (**c**) The projected density of states (PDOS) of pristine TiO${}_{2}$ NT as calculated by means of GLLB-SC exchange–correlation functional within DFT. Vertical black line crossing the DOS plot corresponds to VBM of the pristine TiO${}_{2}$ NT, while red and blue lines correspond to O${}_{2}$/H${}_{2}$O and H${}^{+}$/H${}_{2}$ redox potentials with respect to the vacuum level, respectively. (**d**) is the calculated optical absorption spectrum of the pristine TiO${}_{2}$ NT. Vertical black line crossing the spectrum corresponds to the band gap width of the pristine TiO${}_{2}$ NT.

Figure 1a,b illustrate the front and side projections of the studied NT. The unit cell of pristine anatase (8,0) TiO${}_{2}$ (101) NT was periodically extended 4 times along the *x* axis. Thus, the NT model consists of 12 TiO${}_{2}$ formula units or 96 atoms in total (Figure 1a). To fulfill the conditions for photocatalytic water splitting without the appliance of the external potential, two requirements have to be met [5,14]. The VBM of photocatalyst has to be smaller than the energy of thermodynamically favorable oxygen reduction reaction (−5.67 eV with respect to the vacuum level), while the CBM should be bigger than the energy of standard hydrogen electrode (SHE, −4.44 eV with respect to vacuum level). Figure 1c shows the projected density of states (PDOS) of pristine TiO${}_{2}$ NT. The calculated band gap of the pristine TiO${}_{2}$ NT is 3.257 eV, which is in a good agreement with the titania band gap of ∼3 eV reported elsewhere [39,40,41,42]. The VBM and CBM positions of the pristine TiO${}_{2}$ NT meet the criteria for water splitting under ultraviolet (UV) irradiation. To gain more insight about the utilization of solar light, the calculated absorption spectrum of pristine TiO${}_{2}$ NT is shown in Figure 1d. The wide band gap of the TiO${}_{2}$ NT restricts the efficiency of solar light absorption as only about four percent of the solar spectrum falls in the UV range. In our previous study on N- and S-doped TiO${}_{2}$ NT [15], we predict an obvious blueshift of the imaginary part of the dielectric function. As it is reported in the literature, the absorption spectrum of TiO${}_{2}$ NT can be slightly blueshifted in comparison with TiO${}_{2}$ thin films [39] and powders [40]. The blueshift of the absorption spectrum is well pronounced for the pristine TiO${}_{2}$ NT.

Our group has previously studied the anatase (n,0) TiO${}_{2}$ (001) NT and anatase (0,n) TiO${}_{2}$ (101) NTs [13,14,15,43] by means of ground state total energy calculations. In this work, the pristine TiO${}_{2}$ NT is rolled up from the anatase TiO${}_{2}$ (101) 6-layered slab. The aim of the current study is to understand the influence of substitutional N and S atoms replacing the host oxygens on the improvement of band edge positions in view of enhancement in light absorption. We note that the NT modeled in this study can hardly be synthesized. However, concerning the balance between convergence criteria and structure stability, the elaborated model presents most of the features arising from the substitutional atoms at low computational cost. Our strategy for prediction of the most photocatalytically efficient doped TiO${}_{2}$ NT material can be summarized as follows: (i) The VBM and CBM must meet the conditions for water splitting (VBM < O${}_{2}$/H${}_{2}$O < H${}^{+}$/H${}_{2}$< CBM); (ii) doped TiO${}_{2}$ NT has to be able to absorb maximum photons from solar irradiation in visible range. The absorption spectrum calculated for nanostructures under study is expected to be redshifted and demonstrate obvious enhancement. The mechanism of absorption is represented by calculated TCMs to provide a deeper understanding of electron and hole transition of involved substitutional atoms.

### 3.2. N- and S-Doped TiO${}_{2}$ NT

#### 3.2.1. Ground State

**Figure 2:**
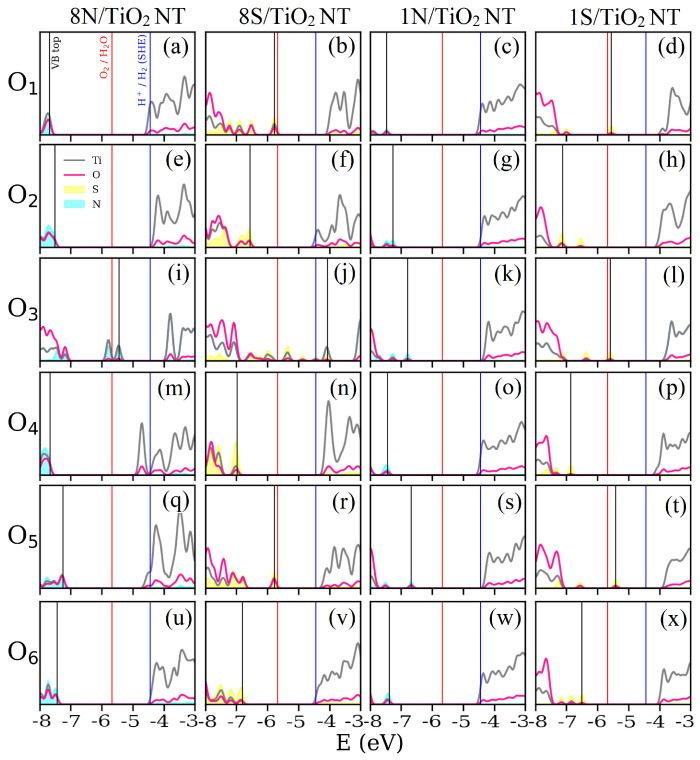
Projected density of states (PDOS) of the N- (cyan) or S- (yellow) doped TiO${}_{2}$ NT. Each row corresponds to the numbered host oxygens substituted by dopant (O${}_{1}$ to O${}_{6}$); each column represents the total number of doped atoms (8N, 8S, 1N, 1S). (**a**) 8N/TiO${}_{2}$ NT at O${}_{1}$; (**b**) 8S/TiO${}_{2}$ NT at O${}_{1}$; (**c**) 1N/TiO${}_{2}$ NT at O${}_{1}$; (**d**) 1S/TiO${}_{2}$ NT at O${}_{1}$; (**e**) 8N/TiO${}_{2}$ NT at O${}_{2}$; (**f**) 8S/TiO${}_{2}$ NT at O${}_{2}$; (**g**) 1N/TiO${}_{2}$ NT at O${}_{2}$; (**h**) 1S/TiO${}_{2}$ NT at O${}_{2}$; (**i**) 8N/TiO${}_{2}$ NT at O${}_{3}$; (**j**) 8S/TiO${}_{2}$ NT at O${}_{3}$; (**k**) 1N/TiO${}_{2}$ NT at O${}_{3}$; (**l**) 1S/TiO${}_{2}$ NT at O${}_{3}$; (**m**) 8N/TiO${}_{2}$ NT at O${}_{4}$; (**n**) 8S/TiO${}_{2}$ NT at O${}_{4}$; (**o**) 1N/TiO${}_{2}$ NT at O${}_{4}$; (**p**) 1S/TiO${}_{2}$ NT at O${}_{4}$; (**q**) 8N/TiO${}_{2}$ NT at O${}_{5}$; (**r**) 8S/TiO${}_{2}$ NT at O${}_{5}$; (**s**) 1N/TiO${}_{2}$ NT at O${}_{5}$; (**t**) 1S/TiO${}_{2}$ NT at O${}_{5}$; (**u**) 8N/TiO${}_{2}$ NT at O${}_{6}$; (**v**) 8S/TiO${}_{2}$ NT at O${}_{6}$; (**w**) 1N/TiO${}_{2}$ NT at O${}_{6}$; (**x**) 1S/TiO${}_{2}$ NT at O${}_{6}$. Vertical lines crossing all the DOS plots correspond to VBM of the doped TiO${}_{2}$ NT (black), while red lines and blue lines correspond to the O${}_{2}$/H${}_{2}$O and H${}^{+}$/H${}_{2}$ redox potentials positioned with respect to the vacuum level, respectively. The PDOS peaks of dopants are scaled by the factor of two.

In this work, two cases of the doped TiO${}_{2}$ NT are considered. In the first case, the substitutional N or S atoms in the periodically repeated unit (inset in the Figure 1a) are replicated along the whole NT wall. As the unit cell of nanotube NT is repeated by 8 rototranslational symmetry operators (i.e., rotation axis is of 8th order), the total number of substitutional atoms is therefore equal to eight. All such dopant combinations are marked as 8N/TiO${}_{2}$ NT and 8S/TiO${}_{2}$ NT, respectively. In the second case, only one N or S atom replaces the numbered oxygen atom in the whole NT wall. These combinations are marked as 1N/TiO${}_{2}$ NT and 1S/TiO${}_{2}$ NT, respectively. Substitutional N and S atoms sequentially replace the numbered host oxygen atoms in six positions depicted in the inset in Figure 1a. Figure 2 shows the projected density of states (PDOS) calculated for the 24 substitutional configurations of the doped TiO${}_{2}$ NT. In total, the S-doped TiO${}_{2}$ NT shows a more general improvement of the band gap width for both 8S/TiO${}_{2}$ NT and 1S/TiO${}_{2}$ NT. According to the electronic configuration of the S dopant, extra electrons contribute to the mid-gap states of the S-doped TiO${}_{2}$ NT. As for the N-doped TiO${}_{2}$ NT, most of the induced states are gathered in the vicinity of the VBM of TiO${}_{2}$ NT. Only few configurations can therefore correspond to the relatively isolated mid-gap states (Figure 2i,k).

Calculated PDOSs prove that the doped atoms on the inner surface of NT wall (O${}_{1}$, O${}_{3}$, and O${}_{5}$) have more influence on the electronic structure due to the distance between the dopant and neighboring host atoms. One of the most apparent cases is the 8S/TiO${}_{2}$ at O${}_{3}$ position in Figure 2j. In this case, the doped S atoms not only improve the band gap, but also induce a series of mid-gap states. Taking into account the band gap width, S and N dopants placed at the O${}_{3}$ position is a suitable choice to overcome the UV drawback of the pristine TiO${}_{2}$ NT. However, dopants placed on the inner surface of the NT wall cause another problem in light of photocatalytic water splitting. Based on the criteria for efficient water splitting, the VBM should be placed below the O${}_{2}$/H${}_{2}$O potential. This requirement is not fulfilled for five dopant positions, as shown in Figure 2d,i,j,l,t. The common point of these five cases is that the positions of the numbered oxygen atoms are located on the inner side of the TiO${}_{2}$ NT wall. Although the concentration of dopants can tune the energy of VBM, this approach is not feasible for the positions shown in Figure 2j,l. In contrast, the doped atoms on the outer surface of the NT wall (O${}_{2}$, O${}_{4}$, and O${}_{6}$) meet all conditions for photocatalytic water splitting. Summing up, doping pristine TiO${}_{2}$ NT with N and S improves the VBM and CBM of the band gap; however, the positions of the substitutional atoms play a significant role in meeting the condition of efficient photocatalytic water splitting.

#### 3.2.2. Optical Property

**Figure 3:**
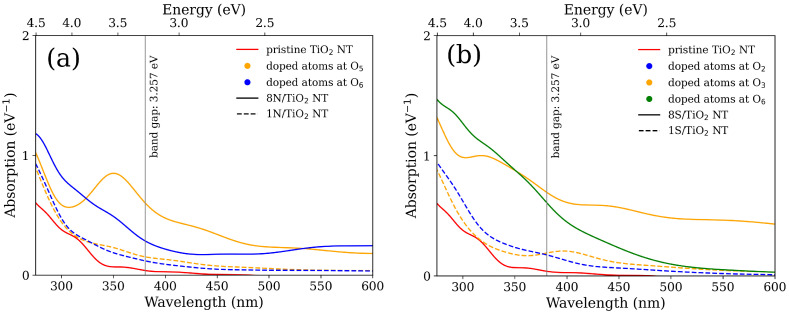
Absorption spectra of (**a**) N- and (**b**) S-doped TiO${}_{2}$ NTs. The red line is the absorption spectrum of the pristine TiO${}_{2}$ NT. The solid lines correspond to the 8N/TiO${}_{2}$- and 8S/TiO${}_{2}$-doped scenario (Figure 2), while the dashed lines are for the single atom doped NTs. For N-doped TiO${}_{2}$ NTs (**a**): the orange color is for the dopant placed at the O${}_{5}$ position and the blue color is for the dopant placed at the O${}_{6}$ position. For S-doped TiO${}_{2}$ NTs (**b**): the orange color is for the dopant placed at O${}_{3}$ position; the blue color is for the dopant placed at O${}_{2}$ position; and the green color is for the dopant placed at O${}_{6}$ position. The orange color is for the dopants placed on the inner surface of the NT wall. The blue and green color are for the dopants placed on the outer surface of the NT wall. The position of O${}_{n}$ is related to the inset of Figure 1a.

To study the influence of N and S dopants on optical photoresponse of the TiO${}_{2}$ NT, in Figure 3, we illustrate the enhancement due to the redshift in optical absorption spectra calculated for the most promising doping scenarios obtained from the ground state calculations shown in Figure 2. The dopants positioned on the inner surface of the NT wall cause strong photon absorption for both N- and S-doped TiO${}_{2}$ NTs. This is an influence of apparent mid-gap states located between the VBM and CBM. For example, the 8S/TiO${}_{2}$ NT doped at O${}_{3}$ position shows the most enhanced absorption as the photon energy is below the band gap of pristine TiO${}_{2}$ NT. This phenomena is observed even if dopant concentration decreases. For the 1S/TiO${}_{2}$ and 1N/TiO${}_{2}$ NT cases doped at O${}_{3}$ position, the absorption is yet to be enhanced. We note that the most obvious photoenhancement and the most pronounced redshift in the calculated absorption spectra depends not only on the positions of dopant, but also can be triggered by the interband transitions at lower energy. The dopants positioned at outer surface of the TiO${}_{2}$ NT wall form more concentrated states in the vicinity of VBM of the pristine TiO${}_{2}$ NT. In spite of the fact that the mid-gap states have tremendous effect on the improvement of the band gap width, they do not always contribute to the enhancement of the optical absorption spectra.

**Figure 4:**
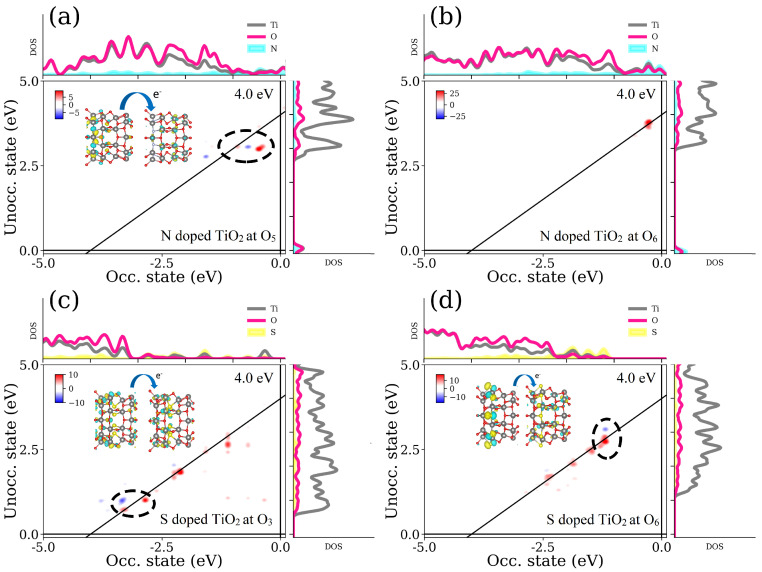
Transition contribution maps (TCM) of N- and S-doped TiO${}_{2}$ NT. (**a**) N-doped TiO${}_{2}$ NT at O${}_{5}$; (**b**) N-doped TiO${}_{2}$ NT at O${}_{6}$; (**c**) S-doped TiO${}_{2}$ NT at O${}_{3}$; (**d**) S-doped TiO${}_{2}$ NT at O${}_{6}$. The diagonal solid-lines are used to emphasize the constant transition energy of 4.0 eV. DOS includes Ti (grey), O (pink), and dopants (N—cyan and S—yellow) projections. The insets show that orbitals contributed to negative transition (marked by ellipse). The reference energy is set to the Fermi level of each nanostructure separately. Below the Fermi level are occupied states; above the Fermi level are unoccupied states. The color scheme set to elucidate the positive (red) and negative (blue) transition contributions. The color intensity is the ratio between S${}_{occ.}^{unocc.}$(ω) and S(ω).

To predict the charge transitions induced by dopants that are placed on both the inner and outer surfaces of NT wall, the calculated TCMs for the electron-hole transitions between occupied and unoccupied states at certain energies (wavelengths) are shown in Figure 4. To be more specific, we focus on the 8N/TiO${}_{2}$ NT and 8S/TiO${}_{2}$ NT doping scenarios to emphasize the transitions at the abundant N or S states. Energy of 4.0 eV with respect to the vacuum level was chosen to elucidate the mechanism of transition and to demonstrate an enhancement of the light absorption if the dopants are placed at outer NT surface. For the N-doped TiO${}_{2}$ NT, it is clear that the dopant placed at inner surface of the TiO${}_{2}$ NT has a negative contribution to absorption, as shown in Figure 4a. In contrast, there is no negative contribution if dopant is placed at outer surface of the TiO${}_{2}$ NT wall, as it is shown in Figure 4b. For the S-doped TiO${}_{2}$ NT, both the dopant placed at inner and outer surfaces of the TiO${}_{2}$ NT wall demonstrate the negative contribution. However, the apparent states gathering around the VBM provide strong positive contribution neglecting the negative part.

From the calculated absorption properties, we can conclude that the mid-gap states positioned between the VBM and the CBM of the pristine TiO${}_{2}$ NT can actually improve the visible-light-driven photoabsorption. In our model, the dopant placed on the inner surface of the TiO${}_{2}$ NT results in the emerging of mid-gap states responsible for the enhanced photoresponse. Our calculations predict that the S-doped titania NT is more promising than the N-doped NT. However, the advantage of defect-induced mid-gap states may not be as attractive considering all the criteria necessary for efficient photocatalytic water splitting. In some doping scenarios, the mid-gap states greatly improving the band gap width do not meet the redox potential condition, i.e., the VBM is bigger than the energy potential of the oxygen reduction reaction or the mid-gap states lead to the negative contribution above the band gap energy. On the other hand, the dopants placed on the outer surface of NT wall with just a few mid-gap states reveal more pronounced redshift at the higher energy.

### 3.3. N+S-Codoped TiO${}_{2}$ NT

**Figure 5:**
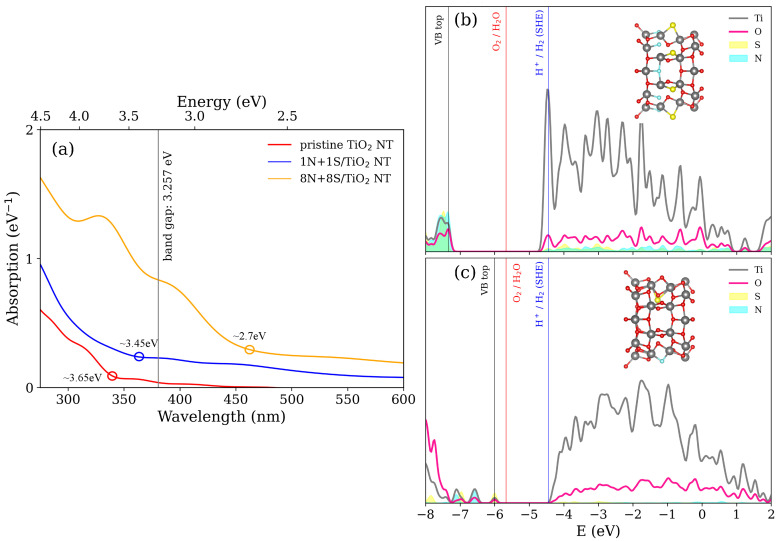
Absorption spectra and calculated projected density of states (PDOS) of N+S-codoped TiO${}_{2}$ NT. (**a**) absorption spectra include pristine TiO${}_{2}$ NT, 8N+8S/TiO${}_{2}$ doped NT, and 1N+1S/TiO${}_{2}$ doped NT. (**b**) PDOS for the 8N+8S/TiO${}_{2}$ NT and (**c**) for the 1N+1S/TiO${}_{2}$ NT. The insets show the equilibrium atomic structures for each model in consideration.

Motivated by the promising results obtained for N- and S-doped NT, we modeled the N+S-codoped TiO${}_{2}$ NT to estimate the most enhanced absorption spectra under the condition necessary for efficient visible-light-driven water splitting. Figure 5 presents the absorption spectra (Figure 5a), calculated PDOS for the 8N+8S/TiO${}_{2}$ NT (Figure 5b), and calculated PDOS for the 1N+1S/TiO${}_{2}$ NT (Figure 5c). For the 8N+8S/TiO${}_{2}$-doped NT, atomic structure is as follows: the N dopants are placed at O${}_{6}$ positions, while the S dopants are placed at O${}_{4}$ positions. The band gap of 8N+8S/TiO${}_{2}$-doped NT is 2.798 eV. The CBM of 8N+8S/TiO${}_{2}$ NT is located at −4.53 eV, which is slightly below SHE equilibrium potential (−4.44 eV). As for the 1N+1S/TiO${}_{2}$-doped NT, its atomic structure consists of the N dopant placed at O${}_{4}$ positions, while the S dopant substitutes host oxygen at O${}_{3}$ positions opposite to the N dopant. The band gap of 1N+1S/TiO${}_{2}$-doped NT is equal to 1.719 eV. We note that the 1S/TiO${}_{2}$-doped NT at O${}_{3}$ position would cause the VBM above the level of equilibrium oxygen reduction reaction potential. Doping of the TiO${}_{2}$ NT with an additional N atom at the suitable position leads not only to the adjustment of the band gap width of the S-doped TiO${}_{2}$ NT, but also restrains the VBM below the redox levels necessary for water splitting. As can be seen from the calculated absorption spectra in Figure 5a, the redshift is well pronounced. The redshift of absorption spectrum means an increase in electron–hole pairs of doped TiO${}_{2}$ NT being involving in the charge transition. In Figure 5a, absorption begins at energies around 3.65 eV (red circle), 3.45 eV (blue circle), and 2.7 eV (orange circle) depicted for pristine TiO${}_{2}$ NT, 1N+1S/TiO${}_{2}$ codoped NT, and 8N+8S/TiO${}_{2}$ codoped NT, respectively. The induced states in the vicinity of the VBM of the pristine TiO${}_{2}$ NT indicate the enhanced interband transition necessary for the increased visible-light-driven photoresponse.

## 4. Conclusions

In this study, we demonstrated that the combination of N and S dopants replacing the host oxygen atoms in TiO${}_{2}$ NT can enhance the visible-light-driven photoresponse and cause redshift of TiO${}_{2}$ NT absorption spectra under the conditions necessary for water splitting. We calculated the electronic and optical properties of N- and S-doped TiO${}_{2}$ NTs, taking into account the high and low dopant ratios by means of DFT and TD-DFT. From our calculations, we can conclude that the shorter the bond length between the dopant and neighbored host atoms, the larger the number of mid-gap states induced. The strong interaction between the dopants and neighboring host atoms leads to the VBM of the doped TiO${}_{2}$ NT positioned above the oxygen redox potential, while the abundant states of doped TiO${}_{2}$ NT are involved in charge transition. These states tend to form the destructive contribution of electron and hole transition at higher energy. We suggest possible scenarios for enhancing the optical absorption of the doped titania NTs allowing for the obvious enhancement and redshift of calculated absorption spectra of doped TiO${}_{2}$ NT depending on dopant concentration. Based on our theoretical calculation, we predict that the use of an N+S-codoped TiO${}_{2}$ NT catalyst could increase the efficiency of visible-light-driven photocatalytic water-splitting applications.

## Data Availability

The raw/processed data required to reproduce these findings cannot be shared at this time as the data also form a part of an ongoing study.
